# Investigating the relationship of positive family history pattern and the incidence and prognosis of idiopathic epilepsy in epilepsy patients

**DOI:** 10.22088/cjim.11.2.219

**Published:** 2020

**Authors:** Masoud Ghiasian, Sajjad Daneshyar, Elham Khanlarzadeh, Mohammadreza Bolouri Novin

**Affiliations:** 1 Department of Neurology, Faculty of Medicine, Hamadan University of Medical Sciences , Hamadan, Iran; 2 Student Research Committee, Hamadan University of Medical Sciences, Hamadan, Iran; 3 Department of Community and Family Medicine, Faculty of Medicine, Hamadan University of Medical Sciences, Hamadan, Iran

**Keywords:** Idiopathic epilepsy, Risk factors, Family history

## Abstract

**Background:**

Epilepsy is one of the most common neurological disorder. This study aimed to investigating the relationship of family history pattern and prognosis of idiopathic epilepsy.

**Methods:**

In this study, 377 patients with epilepsy referring to Imam clinic were investigated. Data were collected by means of a checklist that contained demographic data, age of first seizure attack, response rate to treatment, parental relationship, seizure history, family history and recurrence of seizure. And then the data were analyzed by SPSS Version 23.

**Results:**

Among the 337 patients, 199 (52.8 %) individuals were males and 178 (47.2%) individuals were females. The mean age of patients was 28.3±14 years. Approximately 50% of patients had a history of seizure and epilepsy in one of the close first relatives or relatives who had adequate knowledge of their disease. The mean incidence age of epilepsy was lower in patients with a positive family history of disease than those who did not have a family history of epilepsy (p<0.05). Among the 33 patients who did not respond well to treatment, there was a 75% family history of epilepsy (p<0.05). The average age of epilepsy was lower in those with family marriage, but was not statistically significant.

**Conclusion:**

According to the findings of the present study, the patients diagnosed with idiopathic epilepsy, the family history of epilepsy and seizure, especially in their first degree relatives is fairly high, that may indicate genetic causes in the etiology of the disease.

Aseizure is the physical findings or changes in behavior that occur after an episode of abnormal electrical activity in the brain .Patients with epilepsy are generally at a higher risk of mortality than the general population by two to three times (1, 2). These patients also encounter various types of disabilities and should deal with these disabilities in comparison with the general population. In general, 75% of first seizures are idiopathic. (3, 4) A similar increase in incidence of epilepsy was reported in the first-degree relatives with febrile seizures (5). Epilepsy can arise directly or indirectly from genetic disorders related to a specific gene, a combination of genetics and environmental factors. Approximately, 15% of people with epilepsy have a positive family history (6). Genetic (formerly idiopathic or primary) generalized epilepsies account for 15–20% of all epilepsies (7). The impact of these factors on prognosis of idiopathic epilepsy still remain unknown (8). According to the above-mentioned materials, the present study aimed to determine the relationship between positive family history and the incidence of idiopathic epilepsy in patients with epilepsy.

## Methods

This was a cross-sectional, analytical study. The patients with idiopathic epilepsy visiting Imam Clinic in 2014-2016 were studied. Simple random sampling method was used to select the sample. Accordingly, 377 patients with epilepsy were selected. Inclusion criteria were adult patients with new idiopathic epilepsy; consent to participate in the study and at least 14 years of age. And exclusion criteria were patients with simple seizure or complex partial seizure (with an aura) or history of diabetes, trauma, cancer, radiotherapy, cardiopulmonary or renal disease. The required data were collected using a questionnaire encompassing information on demographic data ,age of the first seizure attack, effective risk factors, family history and other family factors such as cousin marriage in the parents, response rate to treatment and relapse of seizure and other social factors such as occupation and education. Additional required data were collected from the patient’s records available in the clinic including history of the disease, a summary of treatment process, received drugs, results of paraclinical measures for diagnosis, follow-up and response to treatment. The statistical analyses were conducted using SPSS version 23. Continuous variables were summarized as mean±SD or median (IQR) and categorical variables as proportions, *n* (%). Comparison of continuous variables (like age of onset, etc.) in two groups (with and without positive family history) after checking normal distribution was done using student *t*‐test or non-parametric alternative *Mann*-*Whitney U* test. Chi-square test was designed to analyze categorical data (answer to treatment, relapse, etc.) in two groups. A p‐value of <0.05 was considered statistically significant.

## Results

In this study, 377 patients who had been diagnosed with idiopathic epilepsy from 2014 to 2016 were investigated. Of these, 199 (52.8%) were males and 178 (47.2%) were females. The mean age of the patients was 28.3±14 (14-80). 220 (58.4%) lived in rural areas.28 (7.4%) were employees. The demographic characteristics of all patients are shown in table 1. The mean age of the first seizure was 20.79±14.74. A history of epilepsy was reported in the first-degree or the second-degree relatives of 170 (45%) epileptic patients.98 (50.3%) of 195 patients with positive family history reported a history of epilepsy in one of their first-degree relatives (table 2). The mean age of onset of epilepsy in the individuals with a positive family history [18.89 (13.4)] was less than those with no family history of epilepsy [22.71(15.89)]. The difference between these two groups was statistically significant (p=0.013). Of the 33 patients who did not properly respond to treatment, 75% had a family history of epilepsy and 25% had no history of epilepsy. The relationship of incidence and history of epilepsy was also statistically significant (p=0.004).

A positive family history was also reported in 46.15% of the patients with relapse of epilepsy, while no family history of epilepsy was reported in 53.85% of those with relapse of the disease. The difference between these two groups was statistically significant (p=0.004) (table 3).

 110(29.2 %) patient reported consanguineous marriage but relapse, treatment response and age of onset were not statistically different (p=0.88).

**Table 1:** The demographic characteristics of patients with epilepsy

**Demographic characteristics**	**Frequency**
**Gender** Male Female Total	199 (52.8) 178 (47.2) 377 (100)
**Education** Illiterate Less than diploma Diploma, Associate degree Bachelor's degree and higher Total	41 (10.9) 197 (52.2) 104 (27.6) 35 (9.3) 377 (100)
**Residence** Urban Rural Total	157 (41.6) 220 (58.4) 377 (100)
**Marital Status** Married Single Total	150 (39.8) 227 (60.2) 377 (100)
**Job Status** Worker Employee Self-employed Housekeeper Unemployed and other jobs Total	55 (14.6) 28 (7.4) 94 (24.9) 64 (17.0) 136 (36.1) 377 (100)

**Table 2:** Family proportion of epilepsy patients with a positive family history

**Family history**	**Frequency**	**Percentage (%)**
First-degree	98	50.3
Second-degree	72	36.9
Third-degree	7	3.6
Other	18	9.2
Total	195	100

**Table 3:** Relationship between positive family history and relapse of epilepsy in patients

	**with family history** **N (%)**	**without family history** **N (%)**	**Pvalue**
Patient with relapse	90(61.2)	57(38.8)	0.04
Patient without relapse	105(45.6)	125(54.3)	

## Discussion

This descriptive-analytic study aimed to investigate the relationship between positive family history and the incidence and prognosis of idiopathic epilepsy in epileptic patients .The results of this study showed that one second of patients with epilepsy has a history of epilepsy or seizure. This suggests that heredity may be one of the most important risk factors in the etiology of this disease in the studied population. E. Bottachi and M. Leon*et al.* also showed that the incidence of epilepsy in patients with affected parents is 2.5 times higher than those patients with normal parents. The incidence of epilepsy in offspring of the former is 2.4 times higher than the latter (9). Chentouf *et al. *(2015) also showed fourfold-increase in the incidence of the disease in those patients with a positive family history (10). The first step to identify the cause of idiopathic epilepsy is family history of the disease in epileptic patients. Incidence of seizure in the first-degree relatives is an important indicator of involvement of genetic factors in the incidence of the disease (11, 12). BabtainFa.*et al.* showed that one in four epileptic patients has a positive family history (13). The prevalence of this disease in some families is higher than the normal population in some countries due to cousin marriage. Asadi Pouya *et al.* showed that the incidence of epilepsy in the parents with cousin marriage is higher than those with normal parents. Cousin marriage is one of the probable risk factors for epilepsy (14). However, the effect of positive family history and cousin marriage on the diagnosis of idiopathic epilepsy is not determined yet. There are confounding results on the relationship of positive family history of epilepsy and cousin marriage with idiopathic epilepsy (15, 16, 17). Bianchi A.*et al. *(2003) showed that genetic factors are more involved in incidence of epilepsy in Italy (especially idiopathic epilepsy compared to other types of epilepsy) (18).

Findings of the present study showed that male epileptic patients are more than female patients. Previous studies showed that the incidence of epilepsy is slightly higher in males and in those groups with lower socioeconomic status. In this study, 60% of the patients were below diploma. Of the 377 patients, only 28 (7.4%) were employed in the public sector. The rest did not have any specific jobs or were employed in the private sector or had part-time jobs (poor economic and social status). Chen CC.*et al. (2012)* studied on age and gender specific prevalence and incidence of epilepsy showed that male patients had a higher probability of having epilepsy than females (19). The results of this study showed that the age of onset of epilepsy in the patients with a positive family history was four years earlier than those with no history of epilepsy. The difference between these two groups was statistically significant. Epilepsy may occur at any age but the disease may develop at an earlier age in the patients with positive family history than those with no family history of epilepsy (5, 10). CasettaIlaria *et al.* also showed that the incidence of febrile seizure is higher in the patients with a positive family history of the disease, which indicates involvement of genetic factors in the disease (20).

Unfortunately, there is no study on the involvement of genetic factors and family history in patients with idiopathic epilepsy in Iran to compare the results of this study with other studies. Therefore, it is essential to perform further studies in this regard in Iran. Counseling seems necessary to avoid cousin marriage and genetic counseling is recommended for those individuals with a family history of the disease to reduce the incidence of this disease in future generations.

In conclusion** f**indings of the present study showed that incidence of idiopathic epilepsy is higher in the individuals with a family history of epilepsy and seizure, especially in the first degree relatives. This indicates involvement of genetic factors in etiology of the disease. The findings of this study also showed high relapse rates, inappropriate response to treatment and lower age of onset of the disease have a significant relationship with positive family history of epilepsy.
